# Onion Peel Powder as an Antioxidant-Rich Material for Sausages Prepared from Mechanically Separated Fish Meat

**DOI:** 10.3390/antiox9100974

**Published:** 2020-10-11

**Authors:** Jan Bedrníček, Jaromír Kadlec, Ivana Laknerová, Jan Mráz, Eva Samková, Eva Petrášková, Lucie Hasoňová, František Vácha, Vladimír Kron, Pavel Smetana

**Affiliations:** 1 Department of Food Biotechnologies and Agricultural Products’ Quality, Faculty of Agriculture, University of South Bohemia in České Budějovice, Studentská 1668, 370 05 České Budějovice, Czech Republic; kadlec@zf.jcu.cz (J.K.); samkova@zf.jcu.cz (E.S.); hasonova@zf.jcu.cz (L.H.); fvacha@zf.jcu.cz (F.V.); vladimir.kron@trouwnutrition.com (V.K.); smetana@zf.jcu.cz (P.S.); 2 Food Research Institute Prague, Radiová 1285/7, 102 00 Praha 10, Hostivař, Czech Republic; ivana.laknerova@vupp.cz; 3 South Bohemian Research Center of Aquaculture and Biodiversity of Hydrocenoses, Institute of Aquaculture and Protection of Waters, Faculty of Fisheries and Protection of Waters, University of South Bohemia in České Budějovice, Na Sádkách 1780, 370 05 České Budějovice, Czech Republic; jmraz@frov.jcu.cz; 4 Department of Animal Husbandry Sciences, Faculty of Agriculture, University of South Bohemia in České Budějovice, Studentská 1668, 370 05 České Budějovice, Czech Republic; epetraskova@zf.jcu.cz

**Keywords:** mechanically deboned fish meat, onion peel powder, shelf life, fish sausage

## Abstract

Mechanically separated fish meat (MSFM) can be used for the manufacturing of ready-to-eat products, such as sausages; however, it is highly perishable. Several plant by-products, including onion peel, which is rich in polyphenol antioxidants, can be added to food to extend shelf life. This study investigated the effects of the addition of onion peel powder (OPP) to sausage made from MSFM. Sausages were divided into four groups with different amounts of added OPP: 0% (control), 1%, 2%, and 3%. Cooked sausages were stored for 28 days at 5 °C. Samples were analyzed for thiobarbituric acid reactive substances, antioxidant activity, total polyphenol content, pH, and organoleptic properties. The addition of OPP significantly increased antioxidant activity and total polyphenol content and decreased pH, indicating acidic nature of OPP. Polyphenols from OPP effectively suppressed lipid oxidation. A 1–2% addition of OPP enhanced sensory properties. After the 28-day storage, the control samples received the lowest sensory score, due to the presence of a strong fishy odor, which was not present in samples with OPP. HPLC–MS/MS analysis revealed that quercetin is the most dominant compound in OPP. Overall, the results indicate that the addition of OPP in amounts of 1–2% can extend shelf life, without the deterioration of sensory properties.

## 1. Introduction

Fish meat is a nutritionally valuable part of the human diet, and consuming it two times a week is recommended, mostly due to the content of long-chain polyunsaturated n-3 fatty acids with a positive impact on human health [1].

In 2018, about 88% (of total 179 million tons) of fish production was utilized for direct human consumption, primarily in live, fresh, or chilled form; however, the fish industry often extracts only fillets. The remaining 12% was used for non-food purposes, such as fish meal or fish oil [2,3].

Fish processing is closely related to the production of a wide range of waste or by-products. The edible proportion of fish represents approximately 45% of total fish weight; therefore, 55% of the fish can be considered as a waste from processing, including heads, guts, bones, skin, fins, frame, and meat adhered to bones and skin [4]. On the other hand, mechanically separated fish meat (the meat originally adhered to heads, bones, and skin) from commercial fish processing and from fish that is not acceptable, such as fillets and whole fish with non-commercial size, is considered as fish waste, which can be consumed by humans, and, therefore, could be used for the manufacturing of ready-to-eat products [5].

According to Vanhonacker et al. [6], some people may have an aversion to fish consumption. The reason for this is the perceived difficulty in buying, preparing, and cooking; the belief that it is expensive; or due to the unpleasant properties of some varieties of fish, such as the presence of bones and the smell [7]. A ready-to-eat product such as fish sausage could overcome the abovementioned barriers for consumers, and thus might be more easily acceptable.

Unfortunately, fish meat has very short shelf life. A far worse situation is in the case of the utilization of product specific waste. The reasons are high water content, suitable environment for microorganisms, high level of enzymatic activity, and fast oxidation of lipids. The changes start immediately after a fish’s death [8,9].

The incorporation of antioxidants is considered as an effective method to inhibit or delay the lipid oxidation that may result in negative sensory and nutritional changes of meat products, thereby extending the shelf life of products. Despite synthetic antioxidants having been used in recent years, the demand for natural antioxidants has been increasing mainly because of the potentially adverse effects of synthetic antioxidants on human health. Thus, it is becoming increasingly trendy to investigate the effects of natural antioxidants from plant sources (e.g., cereals, fruits, and vegetables) as food products additives [10,11,12,13].

These plant sources could be, for example, vegetable-processing by-products, such as onion peel. Onion (*Allium cepa* L.) is the second most cultivated vegetable in the world, and thousands of tons of waste, generated during its processing, are produced annually just in the European Union [14]. It has been reported that onion peel contains high amounts of dietary fiber, as well as polyphenolic antioxidants, mainly quercetin and its derivatives, which belong to a group of flavonoids [15,16].

Onion flavonoids are well-known for their health benefits, such as antioxidant, anti-inflammatory, antimicrobial, and anticancer effects. In addition, flavonoids play an important role in prevention against oxidative stress, which is a risk factor for development of cardiovascular and neurological diseases [17].

Onion peel or onion-peel extracts (either water or ethanol) have increasingly attracted more attention during the last few years as a functional food ingredient which has been incorporated, for example, into wheat bread [18], gluten-free bread [19], and meat patties [20,21,22], to promote health benefits or prolong product shelf life.

Therefore, the aim of this study was to investigate the effects of the incorporation of onion peel powder (OPP) into a sausage prepared from mechanically separated fish meat on selected chemical, technological, and sensory properties.

## 2. Materials and Methods

### 2.1. Raw Material

Dry and soil-free middle layers of onion peels of the yellow variety Hybelle were donated by a Czech grower of onions (VITAL Czech s.r.o., Všestary, Czech Republic). Onion peels (water content approximately 12%) were ground into a fine onion peel powder (OPP) with particles equal to or smaller than 250 µm and stored at room temperature in the dark until further use. The OPP was then subjected to an analysis of its basic chemical composition, antioxidant activity, polyphenols, and water-holding capacity determination.

Mechanically separated fish meat of the common carp (*Cyprinus carpio* L.) was bought frozen, at −18 °C (freshly produced, only several days old), from a local fish producer (FISH MARKET a.s., České Budějovice, Czech Republic). The separated meat came from fish skeletons (including spine and ribs without head) with adhered meat that remained after filleting. The eggs and pork belly were bought fresh at local markets 1 day prior to the experiment and stored overnight at 5 °C. Several hours before sausage preparation, pork fatback was put into a freezer (−18 °C) to be frozen and easily chopped in a bowl cutter. The spices used for sausage production were purchased from GOLDEN WAY spol. s r. o., Plzeň, Czech Republic. Natural pork casing (diameter 34–36 mm) was purchased from HEROLD ŘEZNICKÉ POTŘEBY s.r.o., Rakovník, Czech Republic.

### 2.2. Preparation of Fish Sausages

Four treatment groups (control and three experimental) were selected to determine the effect of the addition of OPP on the physicochemical and sensory properties of fish–pork sausages. The control group of sausages was prepared according to this recipe (all percentages in control mixture are *w*/*w*): mechanically separated fish meat 49.2%, pork belly 32.8%, garlic 0.4%, salt 1.6%, black pepper 0.2%, caraway seeds 0.1%, marjoram 0.03%, chili pepper 0.2%, paprika 0.5%, egg 2.7%, and ice 12.3%. In the experimental groups, the control mixture was replaced by 1%, 2%, and 3% (*w*/*w*) of OPP (Supplementary Materials Table S1).

The fish and pork meat were chopped into small pieces and weighed separately, according to the recipe. The ingredients were then mixed in a bowl cutter (MTK 661, MADO GmbH, Dornhan, Germany) in the following order: Firstly, pork belly and mechanically separated fish meat were slowly minced with ice. Secondly, salt and spices were added, and, finally, OPP was added at the end of mixing. The total time of mixing of the ingredients was approximately five minutes, and the temperature during this process did not exceed 8 °C. The sausage batter was then stuffed into a natural pork casing. After this, the raw sausages were left for approximately for one hour in the room, to allow the proteins to dissolve. The raw sausages were then cooked in a water bath 1.5 h at 80 °C (to reach internal temperature of 72 °C for 10 min) and then smoked for one hour at 70 °C in a smoking chamber. All batches, after cooling down to 5 °C, were then vacuum-packed and stored at 5 °C for 28 days. Cooking loss was determined during processing. Antioxidant activity, total polyphenol content (TPC), and basic composition chemical composition were measured on the 1st day of storage. The pH, thiobarbituric acid reactive substances (TBARS), CIE L*a*b* color measurement, and microbiological and sensory analysis were assessed on the 1st, 7th, 14th, and 28th day of storage.

### 2.3. Cooking Loss

The sausages were weighted before and after cooking, after cooling down. The cooking loss was calculated according to the following formula:

Cooking loss (%) = [(weight before cooking − weight after cooking)/weight before cooking] × 100

Cooking loss was calculated for each treatment group in triplicate.

### 2.4. Water-Holding Capacity (WHC) of OPP

The water-holding capacity of OPP was determined according to the method described by Benítez et al. [23] with slight modification. Briefly, 1 g of OPP was shaken in a laboratory shaker (180 rpm) in 10 mL of distilled water, at room temperature, for 24 h, in a 15 mL centrifuge tube. The mixture was then centrifuged (2500× *g* for 30 min). The supernatant was transferred to a graduated 10 mL cylinder, where the volume was measured. The result was expressed as mL of H_2_O held by 1 g of OPP. The analysis was conducted in triplicate.

### 2.5. Microbiological Analysis

The prepared fish sausages (before heat treatment, and after heat treatment in, storage days 0, 7, 14, and 28) were aseptically sampled (10 g/sample), mixed with 0.1% peptone (90 mL) in a sterile plastic bag, and homogenized for 1 min, using an electric stomacher (Stomacher 400 Circulator, Fisher Scientific, spol. s r.o., Pardubice, Czech Republic). Serial 10-fold dilutions were prepared from each sample using 1 mL in 0.1% peptone (9 mL). The total viable counts (TVCs) were determined by using the pour plate method according to ISO 4833 [24] and the plates were incubated at 30 °C for 72 h. Horizontal method was used for enumeration of psychrotrophic bacteria [25], and colonies were counted in a solid medium after incubation at 6.5 °C for 10 days. The results were expressed as logarithm of colony forming units per gram of sample (log CFU.g^−1^).

### 2.6. Chemical Analyses

#### 2.6.1. Chemicals

All chemicals, namely quercetin dihydrate (purity ≥ 95%), quercetin-3,4′-*O*-diglucoside (purity ≥ 85%), quercetin-4′-*O*-glucoside (purity ≥ 95%), gallic acid (purity ≥ 99%), sodium acetate (purity ≥ 99%), acetic acid (purity ≥ 99%), sodium acetate (purity ≥ 99%), sodium carbonate, ferric chloride, hydrochloric acid (HCl; 37%), 2,4,4-tris(2-pyridyl)-1,3,5-triazine (TPTZ, purity ≥ 98%), 2,2-diphenyl-1-picrylhydrazyl (DPPH), Trolox (purity ≥ 97%), Trolox (purity ≥ 97%), Folin-Ciocalteau’s phenol reagent, thiobarbituric acid (purity ≥ 98%), butylated hydroxytoluene (BHT; purity ≥ 99%), 1,1,3,3-tetraethoxypropane (TEP), trichloroacetic acid (TCA; purity ≥ 99%), *ortho*-phosphoric acid (PA; purity ≥ 99%), Formic acid (LC/MS grade purity 98–100%), acetonitrile and methanol LC/MS grade, and ethanol for spectroscopy were purchased from Sigma-Aldrich (Prague, Czech Republic).

#### 2.6.2. Basic Chemical Composition of Onion Peel

Water content, ash, crude protein (CP), ether extract (EE), and non-soluble fiber fractions were analyzed in OPP. Moisture was determined by drying the sample, at 105 °C, in an oven, to a constant weight, ash content by combustion at 550 °C, for 16 h in a muffle furnace to obtain light gray ash [26]. Crude protein was determined by the Kjeldahl method, using Velp Kjeltec system UD159 (Mezos spol. s r.o., Hradec Králové, Czech Republic). The amount of nitrogen was multiplied by a factor of 6.25. Lipid content was determined by using an ANKOM XT10 extractor (Ankom Technology, Macedon, NY, USA) with petroleum ether as a solvent. Neutral detergent fiber (NDF), acid detergent fiber (ADF), and acid detergent lignin (ADL) were analyzed according to the modified method of Van Soest et al. [27], using ANKOM A200 Fiber Analyzer (Ankom Technology, Macedon, NY, USA). Afterward, cellulose, hemicellulose, and nitrogen-free extract content were calculated as follows:

Hemicellulose = NDF − ADF

Cellulose = ADF − ADL

Nitrogen-free extract = 100 − (% of water, ash, crude protein, NDF, and ether extract)

All analyses of onion peels were assessed in triplicate.

#### 2.6.3. Basic Chemical Composition of Fortified Fish Sausages

The basic chemical composition of sausages (Supplementary Materials Table S1), namely water, fat, protein, collagen, and salt content were measured, using Fourier transformation near infrared spectroscopy (FT-NIR) instrumentation (FT-NIR Master^TM^ N500; BÜCHI, Flawil, Switzerland), according to the manufacturer’s instructions. Approximately 50 g of sample from each group was homogenized, put into a Petri dish, and analyzed by using the NIR Master^TM^, which scanned samples over an NIR range of 4000–10,000 cm^−1^, with a resolution of 4 cm^−1^. Three independent samples from each group were analyzed.

#### 2.6.4. pH Measurement

The pH of cooked sausages was measured by using a pH meter equipped with a spear-type electrode (Spear-type electrode HC 124, Fisher Scientific, spol. s r.o., Pardubice, Czech Republic). Approximately 25 g of homogenized sample was used to directly measure pH. Three independent samples from each group were measured.

#### 2.6.5. Antioxidant Activity

Two spectrophotometric methods for measurement of antioxidant activity of samples were selected: DPPH assay, to assess free radical scavenging ability, and FRAP assay, to evaluate reducing ability.

##### Extraction of Samples for DPPH and FRAP Analyses

Then, 0.2 g of homogenized sample (either OPP of fish sausages) was extracted in 9.8 mL of 90% methanol (*v*/*v*), shaken in a laboratory shaker for 10 min, and then centrifuged at 7000 RPM, at 5 °C for 15 min. The collected supernatant was further used for DPPH and FRAP (Ferric Reducing Antioxidant Power) analyses. Two independent samples from each treatment groups were taken, and each sample was extracted twice and analyzed (for DPPH and FRAP) in duplicate (*n* = 8/group).

##### DPPH

Antioxidant activity, using DPPH as a reaction agent, was assessed according to Brand-Williams et al. [28] with modifications. To 4 mL of DPPH solution in methanol (27.5 µg/mL), 100 µL of sample extract (either OPP of fish sausages) was added. The mixture of DPPH solution and sample extract was kept for 2 h, in the dark, at room temperature. After this, the absorbance was measured against a blank at 515 nm. The results are expressed as Trolox equivalents (TE)/g FW (fresh weight).

##### FRAP

FRAP was analyzed according to the method described by Dudonné et al. [29], but with slight modification. To prepare FRAP reagent, 100 mL of acetate buffer (300 mM, pH 3.6) was mixed with 10 mL of TPTZ (10 mM) in 40 mM HCl and with 10 mL of 10 mM ferric chloride. Then, a 0.1 mL of sample extract (either OPP or fish sausages) was added to 4 mL of FRAP reagent. The reaction mixture was kept for 30 min, at 37 °C. The absorbance was measured against the acetate buffer (blank) at 593 nm. Results are expressed as TE/g FW.

#### 2.6.6. Total Polyphenol Content

Firstly, 5 g of homogenized (either OPP of fish sausages) was extracted in 100 mL of 80% ethanol (*v*/*v*) under reflux (90 °C/120 min.). A modified method of Lachman et al. [30] was used to determine TPC. An aliquot (1 mL) of filtrate sample extract (as described above) was transferred into a 50 mL volumetric flask. After this, deionized water (10 mL), Folin–Ciocâlteu reagent (2.5 mL) and 20% sodium carbonate (7.5 mL) were added to the sample and properly mixed. Then the flask was filled with deionized water up to the mark. The whole reaction mixture was incubated for 120 min at room temperature, followed by the absorbance measurement at 765 nm. Two independent samples from each treatment group were taken, and each sample was analyzed in duplicate (*n* = 4). The results are expressed as gallic acid equivalents (GAE)/g FW.

#### 2.6.7. HPLC–MS/MS Quantification of Main OPP Flavonols

Extraction and HPLC–MS/MS quantification of main flavonol compounds (quercetin, quercetin-4′-*O*-glucoside, and quercetin-3,4′-*O*-diglucoside) presented in OPP were assessed according to the method described by Bedrníček et al. [19]. Briefly, 0.25 g of OPP was mixed in 5 mL of methanol and extracted in an ultrasonic bath for 15 min and occasionally shaken. The extract was centrifuged at 4000 rpm for 15 min and kept at −18 °C until analysis.

Then, 5 µL of the supernatant was injected into the HPLC–MS/MS system. The analysis was carried out on the HPLC Dionex UltiMate 3000 system coupled with an Agilent 6420 triple-quad mass spectrometer (MS) equipped with an electrospray ionization source. Phenomenex Kinetex C18 column with diameters of 150 × 2.1 mm, (particle size of 2.6 µm), maintained at 35 °C, was used for the separation. Mobile phases consisted of mobile phase A (5% acetonitrile and 0.5% formic acid) and B (100 acetonitrile); the flow rate of mobile phase was 0.2 mL/min. During the separation, mobile phase B linearly increased from 15% to 70% within 20 min, and then decreased back to 15%. The following step was column equilibration for 5 min prior to the next injection. Separated analytes were then introduced into the MS operating in negative mode with the following settings: 11 L/min of drying gas (N_2_), nebulizer pressure of 35 psi, gas temperature 300 °C, capillary voltage −4 kV, and cell acceleration voltage of 7 V. Optimized Fragmentor voltages, collision energy voltages, and transitions were optimized for each compound (Supplementary Materials Table S2). Calibration curve was prepared with 6 concentration levels in the range between 0.1 to 25 µL/mL. The OPP was extracted twice, and each extraction was analyzed twice (*n* = 4). The results are expressed as µg/g of FW.

#### 2.6.8. TBARS Analysis

Products of secondary lipid peroxidation (TBARS) were measured according to the method of Miller [31]. Then, 1 g of homogenized sample (three independent samples from each group) was mixed with 0.2 mL of BHT (0.2 mg/mL in methanol) and 9.1 mL of mixture of TCA/PA (10% TCA in 0.2 M PA), using homogenizer T18 basic Ultra-Turrax (IKA, Staufen, Germany), and then filtrated. To 1.5 mL of filtrate, 1.5 mL of 0.02 M TBA was added. This mixture was then vortexed and heated at 85 °C for 35 min. After heating, the mixture was pipetted on a 96-well plate, and absorbance was measured at 550 nm on a spectrophotometer (Plate Reader AF 2200; Eppendorf AG, Hamburg, Germany). A standard calibration curve was prepared by using TEP, and results were expressed as micrograms of malondialdehyde (MDA) per gram of a sample.

### 2.7. Color Measurement

CIE L*a*b* color measurement was measured, using a ColorEye XTH Spectrophotometer (Gretag Macbeth, New Windsor, NY, USA). Slices were chosen as a representative part of the sausages. Results are expressed in the L*a*b* scale. Results are presented as mean of three independent samples.

### 2.8. Sensory Analysis

The analysis was assessed by 9 trained panelists (5 men and 4 women, aged 25–70 years) from the Faculty of Agriculture, University of South Bohemia, in České Budějovice, who are familiar with the sensory evaluation of meat products. The sausages were heated in a water bath at 70 °C for 10 min prior to sensory analysis and were served warm. Each panelist received approximately 15 g of each sample (randomly marked with a three-digit number) on a plate, at the same time. Appearance, odor, taste, texture, and overall acceptability were evaluated on a 100 mm unstructured abscissa (100 = like extremely; 0 = dislike extremely). The intensity of fishy odor was also assessed on the 28th day of storage (100 = very intensive fishy odor; 0 = no fishy odor). Sensory analysis was conducted in a room (temperature approximately 22 °C) equipped with a table for each panelist and daylight-type bulbs for balanced light. Water and bread were served as a taste neutralizer and were consumed between the judging of samples.

### 2.9. Statistical Analysis

Data were analyzed with the program Statistica CZ 12 (Statsoft CR), using one-way analysis of variance (ANOVA) or two-way ANOVA, using the following model with a fixed effect of percentage of OPP, storage, and interaction OPP × storage:

Y_ijk_ = μ + OPP_i_ + S_j_ + (OPP×S)_k_ + ε_ijk_

where Y_ijk_ is the dependent variables, i.e., pH, TBARS, CIE L*a*b*, sensory characteristics, and cooking loss; μ is the mean; OPP_i_ is the percentage of onion peel powder (i = 4; 0%, 1%, 2%, and 3% of OPP); S_j_ = storage (j = 4; 0, 7, 14 and 28 days), ε_ij_ = residual error. Fisher’s LSD test was used for group comparisons (post hoc test). Pearson’s correlation coefficient was used to estimate the association between percentage of OPP addition and antioxidant activity, and TPC, as well as between CIE L*a*b* color measurement and sensory analysis results.

## 3. Results and Discussion

### 3.1. Chemical Composition of OPP

The basic chemical composition, including total polyphenol content, flavonols, antioxidant activity, and WHC, of OPP is summarized in Table 1. Water accounts for 12.31 ± 0.04% of OPP mass. Thus, the remaining part (87.69%) is dry matter, which is mainly represented by structural carbohydrates (fiber), where cellulose is dominant (27.77%), followed by hemicelluloses (2.97%) and lignin (1.26 ± 0.24%). Our finding is consistent with Choi et al. [14], who also found that cellulose is one of the main structural carbohydrates present in onion peel. However, it is well-known that onion peel contains high amounts (up to 28%) of pectin [16,32], predominantly composed of galacturonic acid, galactose, rhamnose, and arabinose, which are considered as a part of the soluble fraction of fiber [33]. Pectin should then be included in the remaining part of dry matter (nitrogen-free extract) after excluding crude protein, ether extract, and ash. The composition of dietary fiber gives the material specific properties that could be beneficial for consumers for health reasons, but also for manufacturers of meat products, because, depending on the composition, fiber has the ability to bind water and fat, and to create gels in meat products [10].

Crude protein, ether extract and ash content in OPP is 2.41 ± 0.61%, 0.85 ± 0.03%, and 8.06 ± 0.01% of FW, respectively. Benítez et al. [15] reported similar values of crude protein and ash. On the other hand, Negesse et al. [34] measured much lower values not only for crude protein and ash but even for NDF, ADF, lignin, and ether extract.

Main flavonols in OPP were also determined by LC–MS/MS, together with a spectrophotometric determination of TPC. Content of quercetin, quercetin-4′-*O*-glucoside, and quercetin-3,4′-*O*-diglucoside is 4.11 ± 0.15, 3.40 ± 0.08, and 0.63 ± 0.03 mg/g, respectively. Quercetin is the most abundant flavonol in OPP which corresponds to the finding of Prokopov et al. [35], who reported a similar concentration of quercetin which was 3.36 mg/g FW. Regarding quercetin-4′-*O*-glucoside, we found a similar concentration as Suh et al. [36], who also determined this compound in onion peel as the second most dominant with a concentration of 1.9 mg/g FW. Only a small concentration of quercetin-3,4′-*O*-diglucoside was found in OPP. It is a foreseeable result because, according to Takahama and Hirota [37], quercetin is mainly presented in onion peel as aglycone due to the presence of glucosidases, which release quercetin from the glycosidic bond. Other authors, however, found a concentration of total flavonols, represented mainly by quercetin and only to a smaller extent quercetin-4′-*O*-glucoside, in onion peel in the range between 2.6% and 6.5% by weight [38].

The concentration of total polyphenols in OPP is 78.60 ± 1.46 mg GAE/g FW. This value is comparable, but slightly higher, than with Chung et al. [21], who reported a concentration of total polyphenols 69.23 ± 0.44 mg GAE/g DM.

The presence of flavonols in onion peel is tightly related to high antioxidant activity, using DPPH (84.97 ± 2.61 mg TE/g FW) and FRAP method (91.47 ± 2.81 mg TE/g FW). According to Ly et al. [39], quercetin is the most responsible compound for the high antioxidant activity of onion peel, but also glycosidic forms of quercetin contribute significantly to the total antioxidant activity. Jeon et al. [40] also reported that onion peel shows superoxide dismutase-like activity. According to Shah et al. [12], onion peel extract can scavenge O_2_**^•^**^−^ and HO**^•^** radicals. Onion peels are also capable to scavenge free radicals in ORAC (oxygen radical absorbance capacity) antioxidant activity measurement [41].

WHC of dietary fiber is an important technological feature that is important for the incorporation of fiber into a meat product recipe. WHC of OPP was found to be 4.20 ± 0.10 mL/g (Table 1). This value seems to be very low compared to onion fiber concentrates that were analyzed by Benítez et al. [23]. They could absorb much more water (7.9–10.0 mL/g) than OPP. Furthermore, Mehta et al. [42] summarize the WHC of a wide range of plant fibers as being in the range from 2.8 mL/g for wheat bran to 35.4 mL/g for sugar beet fiber. In addition, WHC of a fiber is related to its chemical structure, pH, ionic strength, and particle size. Regarding pH, 1% solution of OPP in deionized water has a value of 4.65 ± 0.06, indicating its acidic nature, since pectin is an acidic heteropolysaccharide with galacturonic acid (with partly nonmethylated carboxyl groups), as a main structural component [43].

Nevertheless, it is very hard to compare the content of various compounds in onion peel with other authors, since different authors use different layers of onion peel that are either closer to or further from the surface of the onion, and could differ, for example, in moisture content. According to Benítez et al. [15] and Cheng et al. [44], the chemical composition and compound proportions change from the inner to outer layers of onion. Another reason could be that the composition is affected by many factors, such as type of cultivar, stage of maturation, environmental and agronomic conditions, and storage time [14].

### 3.2. Cooking Loss

**Figure 1:**
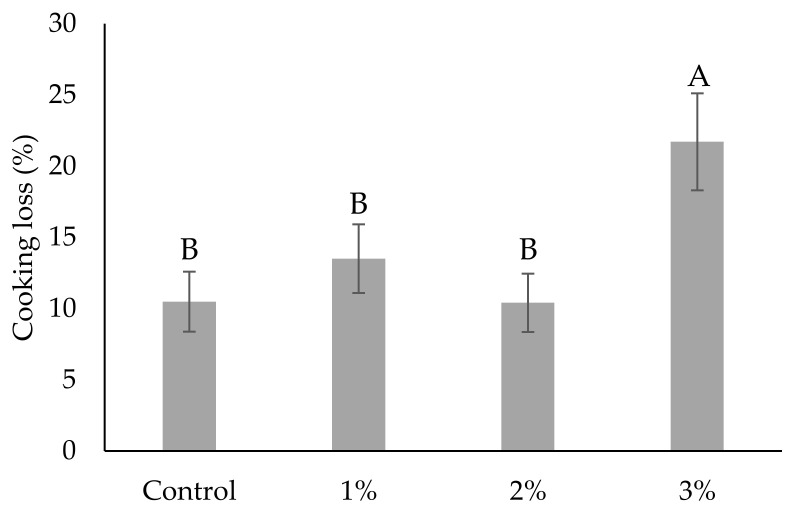
Cooking loss of fish sausages without (control) and with 1%, 2%, and 3% (*w*/*w*) of onion peel powder. ^A,B^ Bars, representing means (*n* = 3), with different letters differ significantly (*p* < 0.05).

In general, weight loss of meat products during heat treatment could be lowered by the addition of plant fiber. According to Ktari et al. [45], cooking method, type of additives, and type and amount of fat affect cooking loss. The influence of the addition of different amounts of OPP on the cooking loss of fish sausages is given in Figure 1. Cooking loss ranged from 10.5% to 21.7%. The lowest value was found paradoxically in samples with no addition of OPP and highest in the group with 3% of OPP. Thus, the 3 % addition of OPP negatively affected this parameter (*p* < 0.05). Control samples did not differ (*p* < 0.05) from samples with 1% and 2% of OPP.

This is very interesting, and the results indicate that a 2% addition of OPP seems to be a threshold in this particular fish sausage recipe and that, after further addition of OPP, cooking loss increases. There are several explanations that could clarify this phenomenon. It could be explained by the degradation of polysaccharides (especially pectin) in OPP during thermal treatment of fish sausages, which can lead to the lowered water-holding capacity of fiber. Another reason could be that the fiber presented in OPP can absorb water much faster than solubilized proteins of meat, but that is not stable at elevated temperatures, and it releases water during cooking and, hence, increases cooking loss. It indicates that the bond between water and fibre is not as strong as between meat protein matrix and water. According to Feiner [46], WHC is also influenced by pH, because a drop of pH lowers the amount of water held by meat. Thus, a very low pH of group with 3% of OPP (Table 2) could negatively affect its ability to hold water during cooking.

Unlike many experiments that resulted in enhanced WHC and/or cooking loss of meat products enriched with plant dietary fiber [47,48], there have been published several papers that reported that the addition of certain types of plant dietary fiber can negatively influence cooking loss [49,50,51,52]. Similar results were obtained by Chung et al. [21], who incorporated 0.3% and 0.6% of onion skin powder into Hanwoo Tteok-galbi (traditional Korean beef patties). Cooking loss was the same or slightly higher compared to control samples. Contrary results, on the other hand, were published by Kurt et al. [22], who added 0%, 1.5%, 3%, and 6% of onion skin powder into cooked chicken meat patties. Cooking loss decreased as the concentration of onion skin powder increased. However, the later mentioned author used a different recipe of meat product (e.g., with chicken meat), a different thermal treatment (180 °C/15 min), and a different particle size of onion skin powder (500 µm vs. ≤250 µm in our study), which could affect results, because, according to Jongaroontaprangsee et al. [53], the particle size of plant dietary fiber powder could alter water absorption.

### 3.3. Antioxidant Activity and TPC of Fish Sausages

Various antioxidants from natural sources have been used to increase the antioxidant activity of meat or meat products, e.g., rosemary extract [9] or red grape pomace [54], in order to improve shelf life, organoleptic properties, or health benefits.

**Figure 2:**
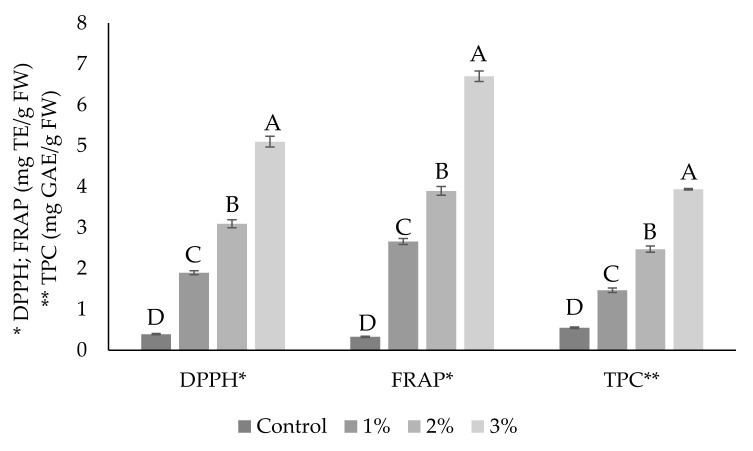
Antioxidant activity (DPPH and FRAP) and total polyphenol content (TPC) of fish sausages without (control) and with 1%, 2%, and 3% (*w*/*w*) of onion peel powder. Bars represent means (*n* = 8 for DPPH and FRAP; *n* = 4 for TPC) ± standard deviation. ^A–D^ Bars within the same analysis with different letters differ significantly (*p* < 0.05).

The addition of OPP into fish sausages had a significantly positive effect (*p* < 0.05) on antioxidant activity (DPPH and FRAP) and TPC, compared to the control (Figure 2). As the content of OPP increased, antioxidant activity, and TPC increased, as well. Thus, the lowest values were observed in the control samples and the highest in samples with 3% of OPP. This is supported by a very strong and statistically significant correlation (*p* < 0.05) which was found between the percentage of OPP in fish sausages and DPPH (*r* = 0.993), FRAP (*r* = 0.991), and TPC (*r* = 0.993) values. It points out that OPP addition contributed significantly to the improvement of antioxidant activity and higher TPC. Despite the fact that no OPP was used in the control group, very low values of antioxidant activity and TPC were found. It could be caused by the presence of other antioxidants occurring in spices used in the recipe. Spices are also source of antioxidant, especially polyphenolic [55]. Increased values of antioxidant activity and TPC in enriched fish sausages indicate that polyphenols in OPP do not decompose completely and at least part of them remains in the finished product. The same trend was already observed in our previous work [20], which focused on onion skin water extracts added into pork patties.

The results show that OPP is a very rich source of antioxidants, and, even at a very low concentration (1%), it can significantly increase the antioxidant activity of certain meat products, especially due to the presence of considerable amounts of quercetin and its derivatives (e.g., glucosides).

### 3.4. Changes in Physicochemical, Microbiological, and Sensory Properties of Fish Sausages during Storage

#### 3.4.1. Physicochemical Properties

Maintaining the stability and superior quality of a meat product during a storage period is of the utmost importance [42]. According to Leistner and Gorris [56], pH is one of the most important factors influencing food preservation and safety. Generally, a reduced pH value suppresses the growth of bacteria, while strong growth is seen in a pH range between 6.2 and 6.4 in different types of meat such as beef, chicken, and pork; however, optimal pH for bacteria growth is seen in a range of 6.1 to 6.7 for fish meat [46]. It was observed that OPP addition significantly (*p* < 0.05) lowered the pH of fortified fish sausages in a dose-dependent manner, compared to the control samples (Table 2). This reduction of pH in samples with OPP could be caused by the acidic nature of OPP, as shown in Table 1 (pH of 1% solution of OPP is 4.65 ± 0.06). As mentioned earlier, onion peels contain high amounts of pectin, primarily composed of galacturonic acid, giving it its acidic nature. The same results were reported by Kurt et al. [22], who incorporated 1.5–6% of onion skin powder into patties. The higher the concentration of onion skin powder, the lower the pH was. On the contrary, Chung et al. [21] obtained different results. By increasing the content of onion skin powder, the pH of Hanwoo Tteok-galbi grew, as well.

Storage time also significantly (*p* < 0.05) affected pH of sausages. The pH of all the sausages continuously decreased during storage with the highest value for the control samples (5.91 ± 0.00) and the lowest for the samples with 3% of OPP (5.56 ± 0.03). This decrease of pH for all the samples might be due to the presence of spoilage lactic acid bacteria [57] that have a suitable environment for growth in vacuum packed meat products. OPP also contributed to this decrease over time, because a significant (*p* < 0.05) interaction between the percentage of OPP incorporation and storage time was found.

The concentration of secondary lipid oxidation products (TBARS) was also determined (Table 2). The addition of OPP significantly (*p* < 0.05) lowered the concentration of MDA. Even on the first day of storage (after cooking), control samples showed signs of oxidation (0.44 ± 0.06 µg MDA/g), compared to sausages with OPP, where the oxidation was suppressed (0.26 ± 0.10–0.32 ± 0.04 µg MDA/g). This oxidation is probably a result of the exposure of fats that are prone to oxidation at high temperatures during cooking. Similarly, this phenomenon was described by Kurt et al. [22] and by our previous work [20].

Storage time also significantly (*p* < 0.05) affected TBARS values. During the 28-day storage period, the concentration of MDA in the control samples grew to values above 0.71 µg MDA/g, while the samples with OPP remained stably low and did not show any increasing trend. Our results are consistent with Kurt et al. [22], Chung et al. [21], and with Bedrníček et al. [20]. In addition, a strong significantly (*p* < 0.05) negative correlation (*r* = −0.703) was found between % of OPP and MDA concentration in all days of storage. All of these facts indicate that OPP is a promising additive for meat products that is effective in the suppression of fat oxidation in meat products, owing its strong antioxidant properties to high amounts of quercetin.

The variation in color other than the expected norm may be due to the physical characteristics of meat, the concentration and chemical state of pigments therein, and the presence of non-meat ingredients [10]. Color analysis, using the CIE L*a*b* system, is summarized in Table 2. OPP addition had a significant effect (*p* < 0.05) on the lightness of fish sausages (L* value). The lightness of fish sausages proportionally decreased with an increasing percentage of OPP, except for the first day. The difference between samples with 1% and 2% of OPP was small; however, the sample with 3% of OPP had a very dark color. Lightness was affected also by storage time (*p* < 0.05). L* values of the control samples and samples with 1% and 2% of OPP changed with only small alterations, but the samples with 3% were increasingly lighter over time. Furthermore, a* value (redness) was affected by OPP (*p* < 0.05). Values on the first day slightly differed (between 15.72 ± 1.30 and 16.91 ± 0.47), but these differences among treatment groups were more obvious in the later days of storage, where these values were lower with the higher addition of OPP. Again, the samples with 1% and 2% of OPP were very similar. It indicates that OPP can cover the typical red color of a meat product. The same trend was also observed in the case of b* value (yellowness), which was also affected by the addition of OPP (*p* < 0.05). This value was also much lower in the samples with OPP. However, this is an unexpected result, to some extent, because our presumption was that the incorporation of OPP would increase the b* value because peels of yellow onion varieties contain quercetin (as mentioned in Section 3.1) which has yellow color [58]. Neither redness nor yellowness was affected by storage time (*p* > 0.05).

#### 3.4.2. Microbiological Analysis

The initial microbial loads in separated fish meat were 5.76 ± 0.02 log CFU.g^−1^ for TVCs and 5.52 ± 0.02 log CFU.g^−1^ for psychrothrophic bacteria. These relatively high amounts could probably influence the microbial levels in fish sausages before heat treatment, which ranged from 5.29 ± 0.04 log CFU.g^−1^ (3% OPP sample) to 6.03 ± 0.04 log CFU.g^−1^ (1% OPP sample) for TVCs, and from 5.66 ± 0.04 log CFU.g^−1^ (control sample) to 7.02 ± 0.03 log CFU.g^−1^ (3% OPP sample) for psychrothrophic bacteria (Supplementary Materials Table S3). Except for meat, microorganisms could also enter into sausage from other ingredients like spices, as well as from the environment, equipment, and handlers during processing [59]. In our case, the addition of onion peel powder may also have played a role in the initial contamination. On the other hand, similar microbial levels were also found in the control sample. Heat treatment was effective for most of the fish sausage samples examined. Microbial levels on the seventh day of storage ranged from 4.77 ± 0.07 log CFU.g^−1^ (control sample) to an uncountable amount (3% OPP sample) for TVCs, and from 6.30 ± 0.10 log CFU.g^−1^ (control sample) to an uncountable amount (3% OPP sample) for psychrothrophic bacteria. These results suggest the development of spore-forming microorganisms. According to Raju et al. [60], fish sausages are considered as an ideal environment for spores and spoilage microorganisms, and heat treatment is not usually effective for all of them. In this context, it should also be taken into account that onion skins are in close contact with the soil, which is known as a rich source of bacterial spores [61]. On the 14th day of storage, the lowest microbial counts were observed in the 2% OPP sample for both indicator groups, TVC (5.39 ± 0.03 log CFU.g^−1^) and psychrotrophic bacteria (5.71 ± 0.02 log CFU.g^−1^). On the 28th day of storage, all examined samples were found as uncountable.

#### 3.4.3. Sensory Analysis

**Figure 3:**
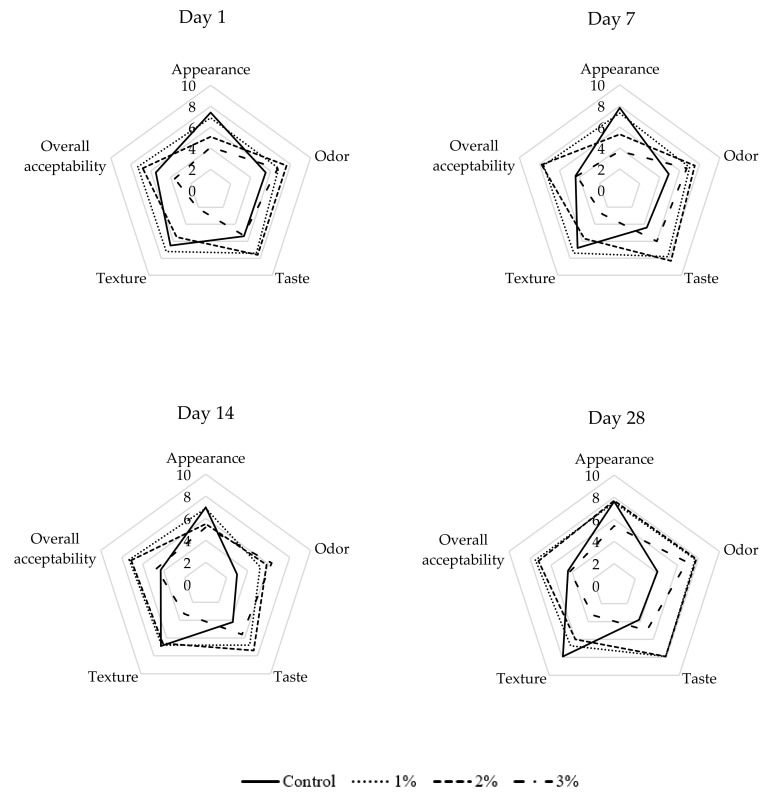
Changes in sensory characteristics of fish sausages without (control) and with 1%, 2%, and 3% (*w*/*w*) of onion peel powder during storage.

Although a diet enhanced by antioxidants may have considerable health effects, sensory properties should not be neglected. Sensory characteristics, particularly taste and appearance, have a great impact on consumers’ preference. The results of the analysis of appearance, odor, taste, texture, and overall acceptability are presented in Figure 3. The intensity of fishy odor on the 28th day of storage is depicted in Supplementary Figure S1. The incorporation of OPP significantly (*p* < 0.05) influenced all evaluated sensory parameters.

It is evident that the addition of more than 1% of OPP negatively influenced the appearance of fish sausage. The addition of OPP caused darker color, compared to the control group (Table 2), which is not usual for meat products. This is supported by a strong correlation coefficient (*r* = 0.762, *p* < 0.05), suggesting that the lighter the sausage is, the more pleasant appearance it has. Together with this, there was also found a significant correlation between appearance and redness (*r* = 0.662; *p* < 0.05). Even though there were slight changes in appearance over time, they were not statistically significant (*p* > 0.05).

The worst score in the case of odor evaluation received the control samples for all days. The addition of OPP positively affected odor pleasantness. Moreover, storage time significantly affected (*p* < 0.05) odor. The pleasantness of odor of control samples continuously decreased over time. This could be related to the presence of fishy odor. It is attributed to compounds including alcohols, aldehydes, ketones, pyrazine, furan and trimethylamine [62]. Furthermore, lipid oxidation is associated with the development of an undesirable fishy odor in fish stored for an extended time [63]. As shown in Supplementary Materials Figure S1, after 28 days of storage, the most intensive fishy odor was perceived in the control group. This means that OPP may cover or inhibit the development of fishy odor. This shows an advantage of utilization of OPP in fish sausages, since some consumers do not like fish products because of the fishy smell [6,7]. The intensity of fishy odor strongly correlates (*r* = 0.999; *p* < 0.001) with the concentration of MDA at 28th day of storage.

Taste is also a very important sensory parameter. The assessors regarded samples with 1% and 2% of OPP as the tastiest samples over the whole storage period. The control samples and samples with 3% of OPP received similar values and did not statistically (*p* < 0.05) differ from each other. The taste score of control samples decreased over time; however, these changes were not significant (*p* > 0.05).

Sensory evaluation showed that the most pleasant texture had the control samples and samples with 1% of OPP. The samples with 2% and 3% of OPP were evaluated very negatively. This study confirms with the previous findings of Kurt et al. [22]. The texture of cooked sausages is usually soft; however, OPP contains considerable amounts of non-soluble fiber (Table 1), mainly represented by cellulose. According to Mehta et al. [42], cellulose is responsible for mechanical strength of food. Harder and less preferred texture is, unfortunately, a consequence of the incorporation of OPP. Storage time had no significant effect on texture (*p* > 0.05).

In general, among all the samples, the sensory panel preferred those with 1% and 2% of OPP. These samples received the highest score of overall acceptability. This is a satisfactory result, since some consumers do not like the typical fish smell [6], which could still be present in the control samples. On the other hand, 3% of OPP addition is also below the acceptability threshold. The results of sensory analysis demonstrate that only a small amount (e.g., 1%) of OPP is able to enhance the palatability of sausages prepared from mechanically separated fish meat.

## 4. Conclusions

Based on our results, it is evident that OPP is able to extend the shelf life of sausages prepared from separated fish meat. The formation of MDA was suppressed by the addition of OPP, and the overall acceptability was also prolonged during storage. Unfortunately, OPP with a particle size ≤250 µm is not an appropriate source of dietary fiber that could enhance the technological properties (such as WHC and cooking loss) of fish sausage, which is closely related to the economical aspect of production. However, this problem could be solved by, for example, the reformulation of the meat product recipe, such as the addition of polyphosphates or potato fiber. Overall, the results of this study can be a useful example of the valorization of fish processing by-product together with onion processing by-product that could show a possible way to prevent the loss of valuable nutrients and biologically active substances from the food chain.

## Figures and Tables

**Table 1:** Basic chemical composition of onion peel powder (OPP).

Compound (Unit)	Concentration
Proximate chemical composition (%)	
Water	12.31 ± 0.04
Crude protein	2.41 ± 0.61
Ether extract	0.85 ± 0.03
Ash	8.06 ± 0.01
Fiber (%)	
NDF	32.00 ± 0.11
ADF	29.03 ± 0.03
ADL (Lignin)	1.26 ± 0.24
Cellulose (=ADF − ADL)	27.77
Hemicellulose (=NDF − ADF)	2.97
Nitrogen-free extract (%)	44.37
Polyphenols (mg/g FW)	
Quercetin	4.11 ± 0.15
Quercetin-4′-*O*-glucoside	3.40 ± 0.08
Quercetin-3,4′-*O*-diglucoside	0.63 ± 0.03
Total polyphenol content *	78.60 ± 1.46
Antioxidant activity (mg TE/g)	
DPPH	84.97 ± 2.61
FRAP	91.47 ± 2.81
Water holding capacity (mL/g)	4.20 ± 0.10
pH (1% solution) **	4.65 ± 0.06

Results are expressed as mean ± standard deviation based on fresh weight; TE = trolox equivalent; NDF = neutral detergent fiber; ADF = acid detergent fiber; ADL = acid detergent lignin; DPPH = 2,2-diphenyl-1-picrylhydrazil; FRAP = ferric reducing antioxidant power; FW = fresh weight. * Expressed as mg Galic acid equivalent/g; ** pH of 1% solution of dissolved onion peel powder in deionized water.

**Table 2:** Changes in pH, TBARS (thiobarbituric acid reactive substances), and color measurement values (L, a, and b) of fish sausages without (control) and with 1%, 2%, and 3% (*w*/*w*) of onion peel powder (OPP) during storage.

OPP (%)	Storage (Days)				*p*		
	1	7	14	28	OPP	Storage	OPP × Storage
pH							
Control	6.29 ± 0.02	6.31 ± 0.01	6.14 ± 0.04	5.91 ± 0.00			
1	6.14 ± 0.02	6.15 ± 0.01	5.95 ± 0.01	5.65 ± 0.02	˂0.001	˂0.001	˂0.001
2	5.94 ± 0.01	5.98 ± 0.02	5.80 ± 0.05	5.58 ± 0.02			
3	5.88 ± 0.00	5.90 ± 0.01	5.84 ± 0.04	5.56 ± 0.03			
TBARS (µg MDA/g FW)							
Control	0.44 ± 0.06	0.75 ± 0.05	0.73 ± 0.33	0.71 ± 0.05			
1	0.26 ± 0.10	0.30 ± 0.01	0.14 ± 0.11	0.30 ± 0.08	˂0.001	0.0464	0.0092
2	0.32 ± 0.04	0.38 ± 0.10	0.31 ± 0.10	0.21 ± 0.05			
3	0.28 ± 0.02	0.28 ± 0.07	0.21 ± 0.03	0.21 ± 0.00			
L-value							
Control	55.34 ± 0.44	56.84 ± 1.04	56.39 ± 0.77	55.82 ± 0.57			
1	48.76 ± 0.69	51.71 ± 0.22	50.36 ± 1.11	49.90 ± 0.17	˂0.001	0.0002	0.1736
2	48.38 ± 0.95	48.60 ± 1.22	48.34 ± 0.33	48.33 ± 0.39			
3	44.18 ± 0.59	46.08 ± 1.90	46.35 ± 0.16	46.15 ± 0.12			
a-value							
Control	16.91 ± 0.47	18.09 ± 0.47	18.80 ± 0.42	19.90 ± 0.78			
1	16.78 ± 0.25	15.70 ± 0.25	15.45 ± 0.43	14.60 ± 0.50	˂0.001	0.3339	˂0.001
2	15.72 ± 1.30	15.49 ± 1.30	16.11 ± 0.75	16.21 ± 1.69			
3	16.58 ± 0.42	14.93 ± 0.42	14.50 ± 0.31	13.19 ± 0.20			
b-value							
Control	31.96 ± 0.62	32.62 ± 0.68	32.90 ± 2.03	33.46 ± 3.33			
1	21.83 ± 0.41	26.61 ± 0.65	26.59 ± 0.61	25.08 ± 0.58	˂0.001	0.6626	0.4891
2	25.04 ± 1.84	24.39 ± 1.68	24.73 ± 1.07	24.43 ± 2.41			
3	22.96 ± 0.41	21.01 ± 0.90	22.49 ± 2.10	21.75 ± 3.30			

Results are expressed as a mean (*n* = 3) ± standard deviation; FW = fresh weight.

## Supplementary Materials

The following are available online at https://www.mdpi.com/2076-3921/9/10/974/s1. Figure S1: Intensity of fishy odor of fish sausages without (control) and with 1%, 2%, and 3% (*w*/*w*) of onion peel powder evaluated on the 28th day of storage. Table S1: Recipes and chemical composition of fish sausages without (control) and with 1%, 2%, and 3% (*w*/*w*) of onion peel powder (OPP). Table S2: Settings of Multiple Reaction Monitoring for quantification of selected flavonoids. Table S3: Total viable counts and counts of psychrotrophic bacteria in fish sausages without (control) and with 1%, 2%, and 3% (*w*/*w*) of onion peel powder (OPP).
