# Supplementary material for: Onion Peel Powder as an Antioxidant-Rich Material for Sausages Prepared from Mechanically Separated Fish Meat
Source: Antioxidants (Basel). 2020 Oct 11;9(10):974. doi: 10.3390/antiox9100974 (PMC7601044; doi:10.3390/antiox9100974)
Supplement: Supplementary file 1 [file antioxidants-09-00974-s001.pdf]

antioxidants

Supplementary Material

Onion Peel Powder as an Antioxidant-Rich Material for Sausages Prepared from Mechanically Separated Fish Meat

Jan Bedrníček [1,*], Jaromír Kadlec [1], Ivana Laknerová [2], Jan Mráz [3], Eva Samková [1], Eva Petrášková [4], Lucie Hasoňová [1], František Vácha [1], Vladimír Kron [1] and Pavel Smetana [1]

[1] Department of Food Biotechnologies and Agricultural Products' Quality, Faculty of Agriculture, University of South Bohemia in České Budějovice, Studentská 1668, 370 05 České Budějovice, Czech Republic; kadlec@zf.jcu.cz (J.K.); samkova@zf.jcu.cz (E.S.); hasonova@zf.jcu.cz (L.H.); fvacha@zf.jcu.cz (F.V.); vladimir.kron@trouwnutrition.com (V.K.); smetana@zf.jcu.cz (P.S.)

[2] Food Research Institute Prague, Radiová 1285/7, 102 00 Praha 10, Hostivař, Czech Republic; ivana.laknerova@vupp.cz

[3] South Bohemian Research Center of Aquaculture and Biodiversity of Hydrocenoses, Institute of Aquaculture and Protection of Waters, Faculty of Fisheries and Protection of Waters, University of South Bohemia in České Budějovice, Na Sádkách 1780, 370 05 České Budějovice, Czech Republic; jmraz@frov.jcu.cz

[4] Department of Animal Husbandry Sciences, Faculty of Agriculture, University of South Bohemia in České Budějovice, Studentská 1668, 370 05 České Budějovice, Czech Republic; epetraskova@zf.jcu.cz

* Correspondence: bedrnj00@zf.jcu.cz; Tel.: +420-721-657-114

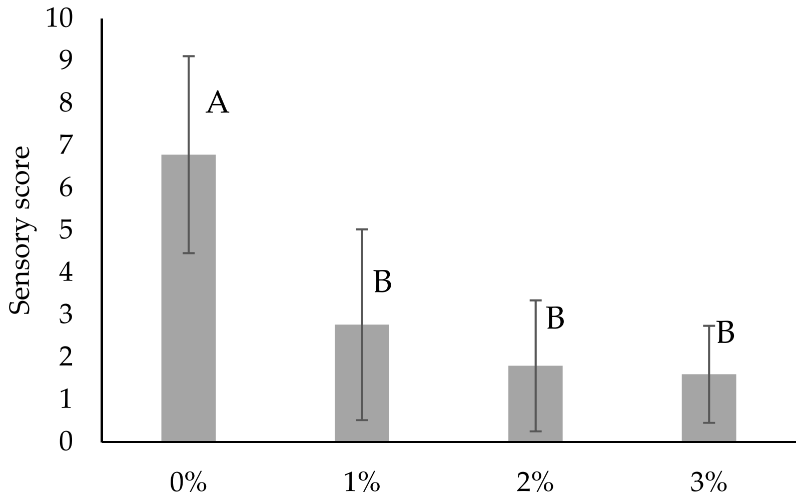

Figure S1. Intensity of fishy odor of fish sausages without (control) and with 1%, 2%, and 3% (*w*/*w*) of onion peel powder evaluated on the 28th day of storage. Bars represent means (n = 9) ± standard deviation; ^{A,B} Bars with different letters differ significantly ($p < 0.05$).

Table S1. Recipes, and chemical composition of fish sausages without (control) and with 1%, 2%, and 3% (*w*/*w*) of onion peel powder.

	**Control**	**Experimental**		
		**1%**	**2%**	**3%**
Ingredients (% w/w)				
Fish meat (mechanically separated)	49.18	48.69	48.20	47.71
Pork belly	32.79	32.46	32.13	31.81
Egg	2.71	2.68	2.65	2.62
Onion peel powder	0	1.00	2.00	3.00
Salt	1.639	1.623	1.607	1.590
Paprika	0.492	0.487	0.482	0.477
Garlic	0.410	0.406	0.402	0.398
Chili pepper	0.205	0.203	0.201	0.199
Black pepper	0.164	0.162	0.161	0.159
Caraway seeds	0.082	0.081	0.080	0.080
Marjoram	0.033	0.032	0.032	0.032
Ice	12.30	12.17	12.05	11.93
Total	100.00	100.00	100.00	100.00
*Chemical composition (%)**				
Moisture	61.83±1.24	63.13±0.04	60.43±0.03	58.99±0.19
Fat	20.15±0.13	19.95±0.02	22.63±0.01	21.62±0.16
Protein	14.30±0.03	13.74±0.03	13.18±0.04	15.36±0.01
Collagen	1.90±0.06	1.53±0.04	1.31±0.04	1.33±0.05
Salt	1.62±0.03	1.51±0.01	1.55±0.01	1.56±0.07

* Results are expressed as mean ± standard deviation (*n* = 3).

Table S2. Settings of Multiple Reaction Monitoring for quantification of selected flavonoids.

**Compound**	**Quantification Transition (*m*/*z*)**	**Fragmentor (V)**	**Collision Energy (eV)**	**Polarity**
Quercetin-3,4′-O-diglucoside	625 → 463	100	10	Negative
Quercetin-4′-O-glucoside	463 → 301	100	10	Negative
Quercetin	301 → 151	130	20	Negative

Table S3. Total viable counts and counts of psychrotrophic bacteria in fish sausages without (control) and with 1%, 2%, and 3% (*w/w*) of onion peel powder (OPP).

**OPP (%)**	**Before Heat Treatment**	**After Heat Treatment**				***p***	
		**Storage (days)**					
		**0**	**7**	**14**	**28**	**OPP**	**Storage**
Total viable counts (log CFU.g^{-1})							
Control	5.89 ± 0.02	<1	4.77 ± 0.07	6.90 ± 0.02	N	0.024	<0.001
1	6.03 ± 0.04	3.06 ± 0.15	6.36 ± 0.05	7.66 ± 0.04	N		
2	5.88 ± 0.01	<1	7.31 ± 0.08	5.39 ± 0.03	N		
3	5.29 ± 0.04	3.18 ± 0.11	N	7.16 ± 0.09	N		
Psychrotrophic bacteria (log CFU.g^{-1})							
Control	5.66 ± 0.04	<1	6.30 ± 0.10	8.48 ± 0.16	N	0.499	<0.001
1	5.70 ± 0.04	<1	6.90 ± 0.01	7.87 ± 0.02	N		
2	6.00 ± 0.05	<1	7.36 ± 0.05	5.71 ± 0.02	N		
3	7.02 ± 0.03	<1	N	7.14 ± 0.15	N		

N-uncountable, CFU- colony forming units; $n = 2$
